# Discovery of *Cytospora* species associated with canker disease of tree hosts from Mount Dongling of China

**DOI:** 10.3897/mycokeys.62.47854

**Published:** 2020-02-03

**Authors:** Haiyan Zhu, Meng Pan, Jadson D.P. Bezerra, Chengming Tian, Xinlei Fan

**Affiliations:** 1 The Key Laboratory for Silviculture and Conservation of Ministry of Education, Beijing Forestry University, Beijing 100083, China Beijing Forestry University Beijing China; 2 State Key Laboratory of Mycology, Institute of Microbiology, Chinese Academy of Sciences, Beijing 100101, China Institute of Microbiology, Chinese Academy of Sciences Beijing China; 3 College of Life Sciences, University of Chinese Academy of Sciences, Beijing 100049, China University of Chinese Academy of Sciences Beijing China; 4 Setor de Micologia, Departamento de Biociências e Tecnologia, Instituto de Patologia Tropical e Saúde Pública (IPTSP), Universidade Federal de Goiás, Rua 235, s/n, Setor Universitário, CEP: 7460505, Goiânia, Goiás, Brazil Universidade Federal de Goiás Goiânia Brazil

**Keywords:** Cytosporaceae, phylogeny, taxonomy, wood-inhabiting fungi

## Abstract

Members of *Cytospora* encompass important plant pathogens, saprobes and endophytes on a wide range of woody hosts with a worldwide distribution. In the current study, we obtained seven representative isolates from six tree hosts of Betulaceae, Juglandaceae, Rosaceae, Tiliaceae and Ulmaceae in Mount Dongling of China. Based on morphological comparison and phylogenetic analyses using partial ITS, LSU, *act*, *rpb2*, *tef1-α* and *tub2* gene sequences, we identified two known species (*Cytospora leucostoma* and *C. pruinopsis*) and two novel species (*C. coryli* and *C. spiraeicola*). These results represent the first study on *Cytospora* species associated with canker disease from Mount Dongling of China.

## Introduction

The genus *Cytospora* was established by Ehrenberg (1818) and belongs to Cytosporaceae, Diaporthales, Sordariomycetes (Wijayawardene et al. 2018, Fan et al. 2020). It is characterised by single or labyrinthine of pycnidial locules, filamentous conidiophores (enteroblastic and phialidic conidiogenous cells) producing hyaline, allantoid conidia in the asexual morph; diaporthalean-like perithecia, clavate to elongate obovoid asci with four or eight hyaline, allantoid ascospores in the sexual morph (Spielman 1983, 1985, Adams et al. 2005). Species of *Cytospora* contain important pathogens that cause stem canker and dieback disease on more than 100 species of woody and coniferous plants, thereby causing severe commercial and ecological damage and significant losses worldwide (Sinclair et al. 1987, Adams et al. 2005, 2006, Fan et al. 2014a, b, 2015a, b, Lawrence et al. 2018, Pan et al. 2018, Zhu et al. 2018a, Zhang et al. 2019). Previous *Cytospora* species and their related sexual morphs viz. *Leucostoma*, *Valsa*, *Valsella* and *Valseutypella* were listed by old fungal literature without any living culture and sufficient evidence for their identification (Fries 1823, Saccardo 1884, Kobayashi 1970, Barr 1978, Sutton 1980, Gvritishvili 1982, Spielman 1983, 1985). Adams et al. (2005) revised the genus *Cytospora* from *Eucalyptus* with 28 species and accepted all sexual genera combined under *Valsa*, either as subgenera or species without additional infrageneric rank. Following the single-name for pleomorphic taxa, *Cytospora* (1818), the older asexual typified name was proposed as the recommended name against *Valsa* (1849), the younger sexual typified name (Fan et al. 2015a, b, Rossman et al. 2015).

Currently, 388 species epithets of *Cytospora* have been recorded in Index Fungorum (2020) (accessed 2 January 2020). However, Kirk et al. (2008) estimated approximately 110 species, but most of them lack herbarium materials, ex-type cultures and DNA sequence data.

Species identification criteria of *Cytospora* were previously carried out by the host-based method and morphology in China; however, these bases are unreliable due to the uninformative illustrations and descriptions, weak host specificity and overlapping morphological characteristics (Teng 1963, Tai 1979, Wei 1979). Recent studies have been able to use multiphase approaches to solve the taxonomy of *Cytospora* (Fan et al. 2014a, b, 2015a, b, Yang et al. 2015, Lawrence et al. 2016, Norphanphoun et al. 2017, Pan et al. 2018, Zhu et al. 2018a, Zhang et al. 2019). Fan et al. (2020) summarised 52 species of *Cytospora* associated with canker and dieback disease in China, using a six gene matrix (ITS, LSU, *act*, *rpb2*, *tef1-α* and *tub2*), of which 13 species were newly introduced.

Mount Dongling has high plant diversity in western Beijing, including more than 1,000 tree hosts (Ma et al. 1995). As more plant species were recorded in this region, the exploration of fungal diversity gradually increased as most fungi are often linked to particular host plants as pathogens or endophytes. Species of *Alternaria*, *Diaporthe*, *Leptostroma*, *Pestalotiopsis* and *Phoma* were the most commonly isolated endophytes from *Pinus tabuliformis* and later, an additional 38 endophytic taxa were identified from *Acer truncatum* from Mount Dongling (Guo et al. 2008, Sun et al. 2011). Further, pathogens belonging in Botryosphaeriales have been identified from Mount Dongling, including five species from *Aplosporella*, *Botryosphaeria* and *Phaeobotryon* (Zhu et al. 2018b). Zhu et al. (2019) subsequently introduced six species of diaporthalean fungi residing in four families (viz. Diaporthaceae, Erythrogloeaceae, Juglanconidaceae and Melanconidaceae) from Mount Dongling. For the current understanding, many common host plants represent high fungal diversity causing canker and dieback disease in Mount Dongling. *Juglans mandshurica* and *J. regia* (Juglandaceae) were infected by *Botryosphaeria dothidea* (Botryosphaeriaceae), *Diaporthe eres*, *D. rostrata* (Diaporthaceae) and *Juglanconis oblonga* (Juglanconidaceae). *Rhus typhina* (Anacardiaceae) was infected by *Aplosporella ginkgonis*, *A. javeedii* (Aplosporellaceae), *Phaeobotryon rhois* and *P. rhoinum* (Botryosphaeriaceae). *Quercus mongolica* (Fagaceae) was infected by *Dendrostoma donglinensis* (Erythrogloeaceae) (Zhu et al. 2018b, 2019).

During the course of cognitive practices to investigate forest pathogens that cause canker or dieback disease in Mount Dongling of China, seven *Cytospora* strains were obtained from six unrelated hosts, i.e. *Corylus mandshurica* (Betulaceae), *Juglans mandshurica* (Juglandaceae), *Prunus sibirica*, *Spiraea salicifolia* (Rosaceae), *Tilia nobilis* (Tiliaceae) and *Ulmus pumila* (Ulmaceae). Phylogenetic analyses inferred from combined ITS, LSU, *act*, *rpb2*, *tef1-α* and *tub2* gene regions were conducted to provide a multi-gene phylogeny for *Cytospora*, based on a large set of freshly collected specimens in Mount Dongling of China. Thus, the current study aims to clarify the systematics and taxonomy of *Cytospora* species with detailed descriptions and illustrations and compare it to known species in the genus.

## Materials and methods

### Sampling and isolation

**Figure 1.**
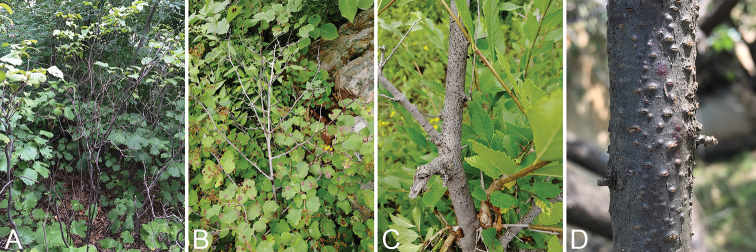
Disease symptoms associated with *Cytospora* species. **A***Corylus mandshurica***B***Spiraea salicifolia***C***Ulmus pumila***D***Prunus sibirica*.

Seven infected branches of six hosts were collected from Mount Dongling of China (Table 1). Sampled trees expressed general symptoms and signs of canker diseases including elongate, slightly sunken and discoloured areas in the bark, several prominent dark sporocarps immersed in bark, erumpent through the surface of bark when mature (Fig. 1). A total of seven isolates was established by removing a mucoid spore mass from conidiomata or ascomata of fresh material, spreading the suspension on the surface of 1.8 % potato dextrose agar (PDA) and incubating at 25 °C for up to 24 h. Single germinating spores were transferred on to fresh PDA plates. Specimens and isolates were deposited in the Key Laboratory for Silviculture and Conservation of the Ministry of Education in Beijing Forestry University (BJFU) and at the working Collection of X.L. Fan (CF), housed at the BJFU. Axenic cultures are maintained in the China Forestry Culture Collection Centre (CFCC).

**Table 1.** Isolates and GenBank accession numbers used in the phylogenetic analyses of *Cytospora*.

Species	Strain^1^	Host	Origin	GenBank accession numbers					
				ITS	LSU	*act*	*rpb2*	*tef1-α*	*tub2*
*Cytospora ailanthicola*	CFCC 89970^T^	*Ailanthus altissima*	Ningxia, China	MH933618	MH933653	MH933526	MH933592	MH933494	MH933565
*Cytospora ampulliformis*	MFLUCC 16-0583^T^	*Sorbus intermedia*	Russia	KY417726	KY417760	KY417692	KY417794	NA	NA
	MFLUCC 16-0629	*Acer platanoides*	Russia	KY417727	KY417761	KY417693	KY417795	NA	NA
*Cytospora amygdali*	CBS 144233^T^	*Prunus dulcis*	California, USA	MG971853	NA	MG972002	NA	MG971659	MG971718
*Cytospora atrocirrhata*	CFCC 89615	*Juglans regia*	Qinghai, China	KR045618	KR045700	KF498673	KU710946	KP310858	KR045659
	CFCC 89616	*Juglans regia*	Qinghai, China	KR045619	KR045701	KF498674	KU710947	KP310859	KR045660
*Cytospora beilinensis*	CFCC 50493^T^	*Pinus armandii*	Beijing, China	MH933619	MH933654	MH933527	NA	MH933495	MH933561
	CFCC 50494	*Pinus armandii*	Beijing, China	MH933620	MH933655	MH933528	NA	MH933496	MH933562
*Cytospora berberidis*	CFCC 89927^T^	*Berberis dasystachya*	Qinghai, China	KR045620	KR045702	KU710990	KU710948	KU710913	KR045661
	CFCC 89933	*Berberis dasystachya*	Qinghai, China	KR045621	KR045703	KU710991	KU710949	KU710914	KR045662
*Cytospora bungeana*	CFCC 50495^T^	*Pinus bungeana*	Shanxi, China	MH933621	MH933656	MH933529	MH933593	MH933497	MH933563
	CFCC 50496	*Pinus bungeana*	Shanxi, China	MH933622	MH933657	MH933530	MH933594	MH933498	MH933564
*Cytospora californica*	CBS 144234^T^	*Juglans regia*	California, USA	MG971935	NA	MG972083	NA	MG971645	NA
*Cytospora carbonacea*	CFCC 89947	*Ulmus pumila*	Qinghai, China	KR045622	KP310812	KP310842	KU710950	KP310855	KP310825
*Cytospora carpobroti*	CMW 48981^T^	*Carpobrotus edulis*	South Africa	MH382812	MH411216	NA	NA	MH411212	MH411207
*Cytospora celtidicola*	CFCC 50497^T^	*Celtis sinensis*	Anhui, China	MH933623	MH933658	MH933531	MH933595	MH933499	MH933566
	CFCC 50498	*Celtis sinensis*	Anhui, China	MH933624	MH933659	MH933532	MH933596	MH933500	MH933567
*Cytospora centrivillosa*	MFLUCC 16-1206^T^	*Sorbus domestica*	Italy	MF190122	MF190068	NA	MF377600	NA	NA
	MFLUCC 17-1660	*Sorbus domestica*	Italy	MF190123	MF190069	NA	MF377601	NA	NA
*Cytospora ceratosperma*	CFCC 89624	*Juglans regia*	Gansu, China	KR045645	KR045724	NA	KU710976	KP310860	KR045686
	CFCC 89625	*Juglans regia*	Gansu, China	KR045646	KR045725	NA	KU710977	KP31086	KR045687
*Cytospora ceratospermopsis*	CFCC 89626^T^	*Juglans regia*	Shaanxi, China	KR045647	KR045726	KU711011	KU710978	KU710934	KR045688
	CFCC 89627	*Juglans regia*	Shaanxi, China	KR045648	KR045727	KU711012	KU710979	KU710935	KR045689
*Cytospora chrysosperma*	CFCC 89629	*Salix psammophila*	Shaanxi, China	KF765673	KF765689	NA	KF765705	NA	NA
	CFCC 89981	Populus alba subsp. pyramidalis	Gansu, China	MH933625	MH933660	MH933533	MH933597	MH933501	MH933568
	CFCC 89982	*Ulmus pumila*	Tibet, China	KP281261	KP310805	KP310835	NA	KP310848	KP310818
***Cytospora coryli***	**CFCC 53162^T^**	***Corylus mandshurica***	**Beijing, China**	**MN854450**	**MN854661**	**NA**	**MN850751**	**MN850758**	**MN861120**
*Cytospora cotini*	MFLUCC 14-1050^T^	*Cotinus coggygria*	Russia	KX430142	KX430143	NA	KX430144	NA	NA
*Cytospora curvata*	MFLUCC 15-0865^T^	*Salix alba*	Russia	KY417728	KY417762	KY417694	KY417796	NA	NA
*Cytospora davidiana*	CXY 1350^T^	*Populus davidiana*	Inner Mongolia, China	KM034870	NA	NA	NA	NA	NA
	CXY 1374	*Populus davidiana*	Heilongjiang, China	KM034869	NA	NA	NA	NA	NA
*Cytospora elaeagni*	CFCC 89632	*Elaeagnus angustifolia*	Ningxia, China	KR045626	KR045706	KU710995	KU710955	KU710918	KR045667
	CFCC 89633	*Elaeagnus angustifolia*	Ningxia, China	KF765677	KF765693	KU710996	KU710956	KU710919	KR045668
*Cytospora elaeagnicola*	CFCC 52882	*Elaeagnus angustifolia*	Xinjiang, China	MK732341	MK732338	MK732344	MK732347	NA	NA
	CFCC 52883	*Elaeagnus angustifolia*	Xinjiang, China	MK732342	MK732339	MK732345	MK732348	NA	NA
	CFCC 52884	*Elaeagnus angustifolia*	Xinjiang, China	MK732343	MK732340	MK732346	MK732349	NA	NA
*Cytospora erumpens*	CFCC 50022	*Prunus padus*	Shanxi, China	MH933627	MH933661	MH933534	NA	MH933502	MH933569
	MFLUCC 16-0580^T^	Salix × fragilis	Russia	KY417733	KY417767	KY417699	KY417801	NA	NA
*Cytospora eucalypti*	CBS 144241	*Eucalyptus globulus*	California, USA	MG971907	NA	MG972056	NA	MG971617	MG971772
*Cytospora euonymicola*	CFCC 50499^T^	*Euonymus kiautschovicus*	Shaanxi, China	MH933628	MH933662	MH933535	MH933598	MH933503	MH933570
	CFCC 50500	*Euonymus kiautschovicus*	Shaanxi, China	MH933629	MH933663	MH933536	MH933599	MH933504	MH933571
*Cytospora euonymina*	CFCC 89993^T^	*Euonymus kiautschovicus*	Shanxi, China	MH933630	MH933664	MH933537	MH933600	MH933505	MH933590
	CFCC 89999	*Euonymus kiautschovicus*	Shanxi, China	MH933631	MH933665	MH933538	MH933601	MH933506	MH933591
*Cytospora fraxinigena*	MFLUCC 14-0868^T^	*Fraxinus ornus*	Italy	MF190133	MF190078	NA	NA	NA	NA
	MFLU 17–0880	*Fraxinus ornus*	Italy	MF190134	MF190079	NA	NA	NA	NA
*Cytospora fugax*	CXY 1371	NA	NA	KM034852	NA	NA	NA	NA	KM034891
	CXY 1381	NA	NA	KM034853	NA	NA	NA	NA	KM034890
*Cytospora gigalocus*	CFCC 89620^T^	*Juglans regia*	Qinghai, China	KR045628	KR045708	KU710997	KU710957	KU710920	KR045669
	CFCC 89621	*Juglans regia*	Qinghai, China	KR045629	KR045709	KU710998	KU710958	KU710921	KR045670
*Cytospora gigaspora*	CFCC 50014	*Juniperus procumbens*	Shanxi, China	KR045630	KR045710	KU710999.	KU710959	KU710922	KR045671
	CFCC 89634^T^	*Salix psammophila*	Shaanxi, China	KF765671	KF765687	KU711000	KU710960	KU710923	KR045672
*Cytospora granati*	CBS 144237^T^	*Punica granatum*	California, USA	MG971799	NA	MG971949	NA	MG971514	MG971664
*Cytospora hippophaës*	CFCC 89639	*Hippophaë rhamnoides*	Gansu, China	KR045632	KR045712	KU711001	KU710961	KU710924	KR045673
	CFCC 89640	*Hippophaë rhamnoides*	Gansu, China	KF765682	KF765698	KF765730	KU710962	KP310865	KR045674
*Cytospora japonica*	CFCC 89956	*Prunus cerasifera*	Ningxia, China	KR045624	KR045704	KU710993	KU710953	KU710916	KR045665
	CFCC 89960	*Prunus cerasifera*	Ningxia, China	KR045625	KR045705	KU710994	KU710954	KU710917	KR045666
*Cytospora joaquinensis*	CBS 144235^T^	*Populus deltoides*	California, USA	MG971895	NA	MG972044	NA	MG971605	MG971761
*Cytospora junipericola*	BBH 42444	*Juniperus communis*	Italy	MF190126	MF190071	NA	NA	MF377579	NA
*Cytospora junipericola*	MFLU 17-0882^T^	*Juniperus communis*	Italy	MF190125	MF190072	NA	NA	MF377580	NA
*Cytospora juniperina*	CFCC 50501^T^	*Juniperus przewalskii*	Sichuan, China	MH933632	MH933666	MH933539	MH933602	MH933507	NA
	CFCC 50502	*Juniperus przewalskii*	Sichuan, China	MH933633	MH933667	MH933540	MH933603	MH933508	MH933572
	CFCC 50503	*Juniperus przewalskii*	Sichuan, China	MH933634	MH933668	MH933541	MH933604	MH933509	NA
*Cytospora kantschavelii*	CXY 1383	*Populus maximowiczii*	Jilin, China	KM034867	NA	NA	NA	NA	NA
	CXY 1386	*Populus maximowiczii*	Chongqing, China	KM034867	NA	NA	NA	NA	NA
*Cytospora leucosperma*	CFCC 89622	*Pyrus bretschneideri*	Gansu, China	KR045616	KR045698	KU710988	KU710944	KU710911	KR045657
	CFCC 89894	*Pyrus bretschneideri*	Qinghai, China	KR045617	KR045699	KU710989	KU710945	KU710912	KR045658
***Cytospora leucostoma***	CFCC 50015	*Sorbus aucuparia*	Ningxia, China	KR045634	KR045714	KU711002	NA	KU710925	KR045675
	CFCC 50016	*Sorbus aucuparia*	Ningxia, China	MH820400	MH820393	MH820408	NA	MH820404	MH820389
	CFCC 50017	*Prunus cerasifera*	Ningxia, China	MH933635	MH933669	MH933542	NA	MH933510	MH933573
	CFCC 50018	*Prunus serrulata*	Gansu, China	MH933636	MH933670	MH933543	NA	MH933511	MH933574
	CFCC 50019	*Rosa helenae*	Gansu, China	MH933637	MH933671	MH933544	NA	NA	NA
	CFCC 50020	*Prunus persica*	Gansu, China	MH933638	MH933672	MH933545	NA	NA	NA
	CFCC 50021	*Prunus salicina*	Gansu, China	MH933639	MH933673	MH933546	NA	MH933512	MH933575
	CFCC 50023	*Cornus alba*	Shanxi, China	KR045635	KR045715	KU711003	KU710964	KU710926	KR045676
	CFCC 50024	*Prunus pseudocerasus*	Qinghai, China	MH933640	MH933674	MH933547	MH933605	NA	MH933576
	CFCC 50467	*Betula platyphylla*	Beijing, China	KT732948	KT732967	NA	NA	NA	NA
	CFCC 50468	*Betula platyphylla*	Beijing, China	KT732949	KT732968	NA	NA	NA	NA
	**CFCC 53140**	***Prunus sibirica***	**Beijing, China**	**MN854445**	**MN854656**	**MN850760**	**MN850746**	**MN850753**	**MN861115**
	**CFCC 53141**	***Prunus sibirica***	**Beijing, China**	**MN854446**	**MN854657**	**MN850761**	**MN850747**	**MN850754**	**MN861116**
	**CFCC 53156**	***Juglans mandshurica***	**Beijing, China**	**MN854447**	**MN854658**	**MN850762**	**MN850748**	**MN850755**	**MN861117**
	MFLUCC 16-0574	*Rosa* sp.	Russia	KY417731	KY417764	KY417696	KY417798	NA	NA
	MFLUCC 16-0589	*Salix alba*	Russia	KY417732	KY417766	KY417698	KY417800	NA	NA
*Cytospora longiostiolata*	MFLUCC 16-0628^T^	Salix × fragilis	Russia	KY417734	KY417768	KY417700	KY417802	NA	NA
*Cytospora longispora*	CBS 144236^T^	*Prunus domestica*	California, USA	MG971905	NA	MG972054	NA	MG971615	MG971764
*Cytospora lumnitzericola*	MFLUCC 17-0508^T^	*Lumnitzera racernosa*	Tailand	MG975778	MH253461	MH253457	MH253453	NA	NA
*Cytospora mali*	CFCC 50028	*Malus pumila*	Gansu, China	MH933641	MH933675	MH933548	MH933606	MH933513	MH933577
	CFCC 50029	*Malus pumila*	Ningxia, China	MH933642	MH933676	MH933549	MH933607	MH933514	MH933578
	CFCC 50030	*Malus pumila*	Shaanxi, China	MH933643	MH933677	MH933550	MH933608	MH933524	MH933579
	CFCC 50031	*Crataegus* sp.	Shanxi, China	KR045636	KR045716	KU711004	KU710965	KU710927	KR045677
	CFCC 50044	*Malus baccata*	Qinghai, China	KR045637	KR045717	KU711005	KU710966	KU710928	KR045678
*Cytospora melnikii*	CFCC 89984	*Rhus typhina*	Xinjiang, China	MH933644	MH933678	MH933551	MH933609	MH933515	MH933580
	MFLUCC 15-0851^T^	*Malus domestica*	Russia	KY417735	KY417769	KY417701	KY417803	NA	NA
	MFLUCC 16-0635	Populus nigra var. italica	Russia	KY417736	KY417770	KY417702	KY417804	NA	NA
*Cytospora nivea*	MFLUCC 15-0860	*Salix acutifolia*	Russia	KY417737	KY417771	KY417703	KY417805	NA	NA
	CFCC 89641	*Elaeagnus angustifolia*	Ningxia, China	KF765683	KF765699	KU711006	KU710967	KU710929	KR045679
	CFCC 89643	*Salix psammophila*	Shaanxi, China	KF765685	KF765701	NA	KU710968	KP310863	KP310829
*Cytospora oleicola*	CBS 144248^T^	*Olea europaea*	California, USA	MG971944	NA	MG972098	NA	MG971660	MG971752
*Cytospora palm*	CXY 1276	*Cotinus coggygria*	Beijing, China	JN402990	NA	NA	NA	KJ781296	NA
	CXY 1280^T^	*Cotinus coggygria*	Beijing, China	JN411939	NA	NA	NA	KJ781297	NA
*Cytospora parakantschavelii*	MFLUCC 15-0857^T^	Populus × sibirica	Russia	KY417738	KY417772	KY417704	KY417806	NA	NA
	MFLUCC 16-0575	*Pyrus pyraster*	Russia	KY417739	KY417773	KY417705	KY417807	NA	NA
*Cytospora parapistaciae*	CBS 144506^T^	*Pistacia vera*	California, USA	MG971804	NA	MG971954	NA	MG971519	MG971669
*Cytospora parasitica*	MFLUCC 15-0507^T^	*Malus domestica*	Russia	KY417740	KY417774	KY417706	KY417808	NA	NA
	XJAU 2542-1	*Malus* sp.	Xinjiang, China	MH798884	MH798897	NA	NA	MH813452	NA
*Cytospora paratranslucens*	MFLUCC 15-0506^T^	Populus alba var. bolleana	Russia	KY417741	KY417775	KY417707	KY417809	NA	NA
	MFLUCC 16-0627	*Populus alba*	Russia	KY417742	KY417776	KY417708	KY417810	NA	NA
*Cytospora pistaciae*	CBS 144238^T^	*Pistacia vera*	California, USA	MG971802	NA	MG971952	NA	MG971517	MG971667
*Cytospora platanicola*	MFLU 17-0327^T^	*Platanus hybrida*	Italy	MH253451	MH253452	MH253449	MH253450	NA	NA
*Cytospora platyclada*	CFCC 50504^T^	*Platycladus orientalis*	Yunnan, China	MH933645	MH933679	MH933552	MH933610	MH933516	MH933581
	CFCC 50505	*Platycladus orientalis*	Yunnan, China	MH933646	MH933680	MH933553	MH933611	MH933517	MH933582
	CFCC 50506	*Platycladus orientalis*	Yunnan, China	MH933647	MH933681	MH933554	MH933612	MH933518	MH933583
*Cytospora platycladicola*	CFCC 50038^T^	*Platycladus orientalis*	Gansu, China	KT222840	MH933682	MH933555	MH933613	MH933519	MH933584
	CFCC 50039	*Platycladus orientalis*	Gansu, China	KR045642	KR045721	KU711008	KU710973	KU710931	KR045683
*Cytospora plurivora*	CBS 144239^T^	*Olea europaea*	California, USA	MG971861	NA	MG972010	NA	MG971572	MG971726
*Cytospora populicola*	CBS 144240^T^	*Populus deltoides*	California, USA	MG971891	NA	MG972040	NA	MG971601	MG971757
*Cytospora populina*	CFCC 89644^T^	*Salix psammophila*	Shaanxi, China	KF765686	KF765702	KU711007	KU710969	KU710930	KR045681
*Cytospora populinopsis*	CFCC 50032^T^	*Sorbus aucuparia*	Ningxia, China	MH933648	MH933683	MH933556	MH933614	MH933520	MH933585
	CFCC 50033	*Sorbus aucuparia*	Ningxia, China	MH933649	MH933684	MH933557	MH933615	MH933521	MH933586
***Cytospora pruinopsis***	CFCC 50034^T^	*Ulmus pumila*	Shaanxi, China	KP281259	KP310806	KP310836	KU710970	KP310849	KP310819
	CFCC 50035	*Ulmus pumila*	Jilin, China	KP281260	KP310807	KP310837	KU710971	KP310850	KP310820
	**CFCC 53153**	***Ulmus pumila***	**Beijing, China**	**MN854451**	**MN854662**	**MN850763**	**MN850752**	**MN850759**	**MN861121**
*Cytospora predappioensis*	MFLUCC 17-2458**^T^**	*Platanus hybrida*	Italy	MG873484	MG873480	NA	NA	NA	NA
*Cytospora pruinosa*	CFCC 50036	*Syringa oblata*	Qinghai, China	KP310800	KP310802	KP310832	NA	KP310845	KP310815
	CFCC 50037	*Syringa oblata*	Qinghai, China	MH933650	MH933685	MH933558	NA	MH933522	MH933589
*Cytospora prunicola*	MFLU 17-0995**^T^**	*Prunus* sp.	Italy	MG742350	MG742351	MG742353	MG742352	NA	NA
*Cytospora punicae*	CBS 144244	*Punica granatum*	California, USA	MG971943	NA	MG972091	NA	MG971654	MG971798
*Cytospora quercicola*	MFLU 17-0881	*Quercus* sp.	Italy	MF190128	MF190074	NA	NA	NA	NA
	MFLUCC 14-0867^T^	*Quercus* sp.	Italy	MF190129	MF190073	NA	NA	NA	NA
*Cytospora ribis*	CFCC 50026	*Ulmus pumila*	Qinghai, China	KP281267	KP310813	KP310843	KU710972	KP310856	KP310826
	CFCC 50027	*Ulmus pumila*	Qinghai, China	KP281268	KP310814	KP310844	NA	KP310857	KP310827
*Cytospora rosae*	MFLU 17-0885	*Rosa canina*	Italy	MF190131	MF190076	NA	NA	NA	NA
*Cytospora rostrata*	CFCC 89909^T^	*Salix cupularis*	Gansu, China	KR045643	KR045722	KU711009	KU710974	KU710932	KR045684
	CFCC 89910	*Salix cupularis*	Gansu, China	KR045644	KR045723	KU711010	KU710975	KU710933	NA
*Cytospora rusanovii*	MFLUCC 15-0853	Populus × sibirica	Russia	KY417743	KY417777	KY417709	KY417811	NA	NA
	MFLUCC 15-0854^T^	*Salix babylonica*	Russia	KY417744	KY417778	KY417710	KY417812	NA	NA
*Cytospora salicacearum*	MFLUCC 15-0861	Salix × fragilis	Russia	KY417745	KY417779	KY417711	KY417813	NA	NA
	MFLUCC 15-0509^T^	*Salix alba*	Russia	KY417746	KY417780	KY417712	KY417814	NA	NA
	MFLUCC 16-0576	Populus nigra var. italica	Russia	KY417741	KY417775	KY417707	KY417809	NA	NA
	MFLUCC 16-0587	*Prunus cerasus*	Russia	KY417742	KY417776	KY417708	KY417810	NA	NA
*Cytospora salicicola*	MFLUCC 15-0866	*Salix alba*	Russia	KY417749	KY417783	KY417715	KY417817	NA	NA
	MFLUCC 14-1052^T^	*Salix alba*	Russia	KU982636	KU982635	KU982637	NA	NA	NA
*Cytospora salicina*	MFLUCC 15-0862^T^	*Salix alba*	Russia	KY417750	KY417784	KY417716	KY417818	NA	NA
	MFLUCC 16-0637	Salix × fragilis	Russia	KY417751	KY417785	KY417717	KY417819	NA	NA
*Cytospora schulzeri*	CFCC 50040	*Malus domestica*	Ningxia, China	KR045649	KR045728	KU711013	KU710980	KU710936	KR045690
	CFCC 50042	*Malus asiatica*	Qinghai, China	KR045650	KR045729	KU711014	KU710981	KU710937	KR045691
*Cytospora sibiraeae*	CFCC 50045^T^	*Sibiraea angustata*	Gansu, China	KR045651	KR045730	KU711015	KU710982	KU710938	KR045692
	CFCC 50046	*Sibiraea angustata*	Gansu, China	KR045652	KR045731	KU711015	KU710983	KU710939	KR045693
*Cytospora sophorae*	CFCC 50047	*Styphnolobium japonicum*	Shanxi, China	KR045653	KR045732	KU711017	KU710984	KU710940	KR045694
	CFCC 50048	*Magnolia grandiflora*	Shanxi, China	MH820401	MH820394	MH820409	MH820397	MH820405	MH820390
	CFCC 89598	*Styphnolobium japonicum*	Gansu, China	KR045654	KR045733	KU711018	KU710985	KU710941	KR045695
*Cytospora sophoricola*	CFCC 89595^T^	Styphnolobium japonicum var. pendula	Gansu, China	KR045655	KR045734	KU711019	KU710986	KU710942	KR045696
	CFCC 89596	Styphnolobium japonicum var. pendula	Gansu, China	KR045656	KR045735	KU711020	KU710987	KU710943	KR045697
*Cytospora sophoriopsis*	CFCC 89600^T^	*Styphnolobium japonicum*	Gansu, China	KR045623	KP310804	KU710992	KU710951	KU710915	KP310817
*Cytospora sorbi*	MFLUCC 16-0631^T^	*Sorbus aucuparia*	Russia	KY417752	KY417786	KY417718	KY417820	NA	NA
*Cytospora sorbicola*	MFLUCC 16-0584^T^	*Acer pseudoplatanus*	Russia	KY417755	KY417789	KY417721	KY417823	NA	NA
	MFLUCC 16-0633	*Cotoneaster melanocarpus*	Russia	KY417758	KY417792	KY417724	KY417826	NA	NA
*Cytospora spiraeae*	CFCC 50049^T^	*Spiraea salicifolia*	Gansu, China	MG707859	MG707643	MG708196	MG708199	NA	NA
	CFCC 50050	*Spiraea salicifolia*	Gansu, China	MG707860	MG707644	MG708197	MG708200	NA	NA
***Cytospora spiraeicola***	**CFCC 53138^T^**	***Spiraea salicifolia***	**Beijing, China**	**MN854448**	**MN854659**	**NA**	**MN850749**	**MN850756**	**MN861118**
	**CFCC 53139**	***Tilia nobilis***	**Beijing, China**	**MN854449**	**MN854660**	**NA**	**MN850750**	**MN850757**	**MN861119**
*Cytospora tamaricicola*	CFCC 50507	*Rosa multifolora*	Yunnan, China	MH933651	MH933686	MH933559	MH933616	MH933525	MH933587
	CFCC 50508^T^	*Tamarix chinensis*	Yunnan, China	MH933652	MH933687	MH933560	MH933617	MH933523	MH933588
*Cytospora tanaitica*	MFLUCC 14-1057^T^	*Betula pubescens*	Russia	KT459411	KT459412	KT459413	NA	NA	NA
*Cytospora thailandica*	MFLUCC 17-0262^T^	*Xylocarpus moluccensis*	Thailand	MG975776	MH253463	MH253459	MH253455	NA	NA
	MFLUCC 17-0263^T^	*Xylocarpus moluccensis*	Thailand	MG975777	MH253464	MH253460	MH253456	NA	NA
*Cytospora tibouchinae*	CPC 26333^T^	*Tibouchina semidecandra*	France	KX228284	KX228335	NA	NA	NA	NA
*Cytospora translucens*	CXY 1351	*Populus davidiana*	Inner Mongolia, China	KM034874	NA	NA	NA	NA	KM034895
*Cytospora ulmi*	MFLUCC 15-0863^T^	*Ulmus minor*	Russia	KY417759	NA	NA	NA	NA	NA
*Cytospora vinacea*	CBS 141585^T^	*Vitis interspecific* hybrid ‘Vidal’	USA	KX256256	NA	NA	NA	KX256277	KX256235
*Cytospora viticola*	CBS 141586^T^	*Vitis vinifera* ‘Cabernet Franc’	USA	KX256239	NA	NA	NA	KX256260	KX256218
*Cytospora xylocarpi*	MFLUCC 17-0251^T^	*Xylocarpus granatum*	Thailand	MG975775	MH253462	MH253458	MH253454	NA	NA
*Diaporthe vaccinii*	CBS 160.32	*Vaccinium macrocarpon*	USA	KC343228	NA	JQ807297	NA	KC343954	KC344196

Abbreviations: **BBH**: BIOTEC Bangkok Herbarium, National Science and Technology Development Agency, Thailand; **CBS**: Westerdijk Fungal Biodiversity Institute (CBS-KNAW Fungal Biodiversity Centre), Utrecht, The Netherlands; **CFCC**: China Forestry Culture Collection Centre, Beijing, China; **CMW**: Culture collection of Michael Wingfield, university of Pretoria, South africa; **CPC**: Culture collection of Pedro Crous, The Netherlands; **MFLU**: Mae Fah Luang University herbarium, Thailand; **MFLUCC**: Mae Fah Luang University Culture Collection, Thailand; **XJAU**: Xinjiang Agricultural University, Xinjiang, China; NA: not applicable. All the new isolates used in this study are indicated in bold type and the strains from generic type species are marked by a superscript (T).

### Morphological analysis

Species identification was based on morphological features of the ascomata or conidiomata from infected host materials and micromorphology, supplemented by cultural characteristics. Microscopic photographs (structure and size of stromata; structure and size of ectostromatic disc and ostioles) were captured using a Leica stereomicroscope (M205 FA) (Leica Microsystems, Wetzlar, Germany). Microscopic observations (shape and size of conidiophores, asci and conidia/ascospores) were determined under a Nikon Eclipse 80i microscope (Nikon Corporation, Tokyo, Japan), equipped with a Nikon digital sight DS-Ri2 high definition colour camera, using differential interference contrast (DIC) illumination. The Nikon software NIS-Elements D Package v. 3.00, Adobe Bridge CS v. 6 and Adobe Photoshop CS v. 5 were used for the manual editing. More than 10 conidiomata/ascomata, 10 asci and 30 conidia/ascospores were measured by Nikon software NIS-Elements D Package v. 3.00 to calculate the mean size/length and respective standard deviations (SD). Colony diameters were measured and the colony features were described using the colour charts of Rayner (1970).

### DNA extraction, PCR amplification and sequencing

Fungal mycelium grown on the cellophane of PDA was scraped for the extraction of genomic DNA following a modified CTAB method (Doyle and Doyle 1990). The primers and PCR conditions are listed in Table 2. DNA sequencing was performed using an ABI PRISM 3730XL DNA Analyser with a BigDye Terminater Kit v.3.1 (Invitrogen, USA) at the Shanghai Invitrogen Biological Technology Company Limited (Beijing, China). The DNA sequences, obtained from forward and reverse primers, were combined using SeqMan v. 7.1.0 in the DNASTAR Lasergene Core Suite software (DNASTAR Inc., Madison, WI, USA).

**Table 2.** Genes used in this study with PCR primers, primer DNA sequence, optimal annealing temperature and corresponding references.

Locus	Definition	Primers	Primer DNA sequence (5'–3')	Optimal annealing temp (°C)	References of primers used
ITS	internal transcribed spacer of ribosomal RNA	ITS1	TCCGTAGGTGAACCTGCGG	51	White et al. 1990
		ITS4	TCCTCCGCTTTTGATATGC		
LSU	large subunit of ribosomal RNA	LROR	ACCCGCTGAACTTAAGC	55	Vilgalys and Hester 1990
		LR7	TACTACCACCAAGATCT		
*act*	actin	ACT-512F	ATGTGCAAGGCCGGTTTCGC	61	Carbone and Kohn 1999
		ACT-783R	TACGAGTCCTTCTGGCCCAT		
*rpb2*	RNA polymerase II second largest subunit	RPB2-5F	GA(T/C)GA(T/C)(A/C)G(A/T)GATCA(T/C)TT(T/C)GG	52	Liu et al. 1999
		RPB2-7cR	CCCAT(A/G)GCTTG(T/C)TT(A/G)CCCAT		
*tef-1α*	translation elongation factor 1-alpha	EF1-668F	CGGTCACTTGATCTACAAGTGC	55	Alves et al. 2008
		EF1-1251R	CCTCGAACTCACCAGTACCG		
*tub2*	beta-tubulin	Bt2a	GGTAACCAAATCGGTGCTGCTTTG	55	Glass and Donaldson 1995
		Bt2b	ACCCTCAGTGTAGTGACCCTTGGC		

### Phylogenetic analyses

The current isolates were initially identified as *Cytospora* species, based on both morphological observations and BLAST results. To clarify their further phylogenetic position, an analysis, based on the combined six genes (ITS, LSU, *act*, *rpb2*, *tef1-α* and *tub2*), was performed to compare *Cytospora* species from the current study with other strains in GenBank. *Diaporthe vaccinii* was selected as the outgroup in all analyses. Subsequent alignments for each gene were generated using MAFFT v.7 (Katoh and Standley 2013) and manually adjusted using MEGA v. 6 (Tamura et al. 2013). Ambiguously aligned sequences were excluded from analysis. Reference sequences were selected, based on ex-type or ex-epitype sequences available from recently published literature (Fan et al. 2014a, b, 2015a, b, 2020, Yang et al. 2015, Lawrence et al. 2016, Norphanphoun et al. 2017, Zhu et al. 2018a, Zhang et al. 2019, Fan et al. 2020) (Table 1). Phylogenetic analyses were performed with PAUP v.4.0b10 for the maximum parsimony (MP) method (Swofford 2003), MrBayes v.3.1.2 for the Bayesian Inference (BI) method (Ronquist and Huelsenbeck 2003) and RAxML for the maximum likelihood (ML) method (Stamatakis 2006).

A partition homogeneity test (PHT) with heuristic search and 1,000 replicates was performed using PAUP v.4.0b10 to test the discrepancy amongst the ITS, LSU, *act*, *rpb2*, *tef1-α* and *tub2* sequence datasets in reconstructing phylogenetic trees. MP analysis was performed using a heuristic search option of 1,000 random-addition sequences with a tree bisection and reconnection (TBR) branch swapping algorithm (Swofford 2003). The branches of zero length were collapsed and all equally parsimonious trees were saved. Clade stability was assessed with a bootstrap analysis of 1,000 replicates (Hillis and Bull 1993). Other parsimony scores, such as tree length (TL), consistency index (CI), retention index (RI) and rescaled consistency (RC), were calculated (Swofford 2003). ML analysis was performed with the GTR + G + I model of site substitution following recent studies (Zhu et al. 2018a), including estimation of gamma-distributed rate heterogeneity and a proportion of invariant sites using PhyML v. 3.0 (Guindon et al. 2010). The branch support was evaluated with a bootstrapping method of 1,000 replicates (Hillis and Bull 1993). BI analysis was performed using a Markov Chain Monte Carlo (MCMC) algorithm with Bayesian posterior probabilities (Rannala and Yang 1996). A nucleotide substitution model was estimated by MrModeltest v.2.3 (Posada and Crandall 1998) and a weighted Bayesian analysis was considered. Two MCMC chains were run from random trees for 1,000,000 generations and trees were sampled each 100 generations. The first 25% of trees were discarded as the burn-in phase of each analysis and the posterior probabilities (BPP) were calculated to assess the remaining trees (Rannala and Yang 1996). The branch support from MP and ML analysis was evaluated with a bootstrapping (BS) method of 1,000 replicates (Hillis and Bull 1993). Phylograms were plotted in Figtree v. 1.4.4 (http://tree.bio.ed.ac.uk/software/figtree) and edited in Adobe Illustrator CS6 v.16.0.0 (https://www.adobe.com/cn/products/illustrator.html). Novel sequences, generated in the current study, were deposited in GenBank (Table 1) and the aligned matrices, used for phylogenetic analyses, were submitted in TreeBASE (www.treebase.org; study ID S25564).

## Results

### Phylogenetic analyses

**Figure 2.**
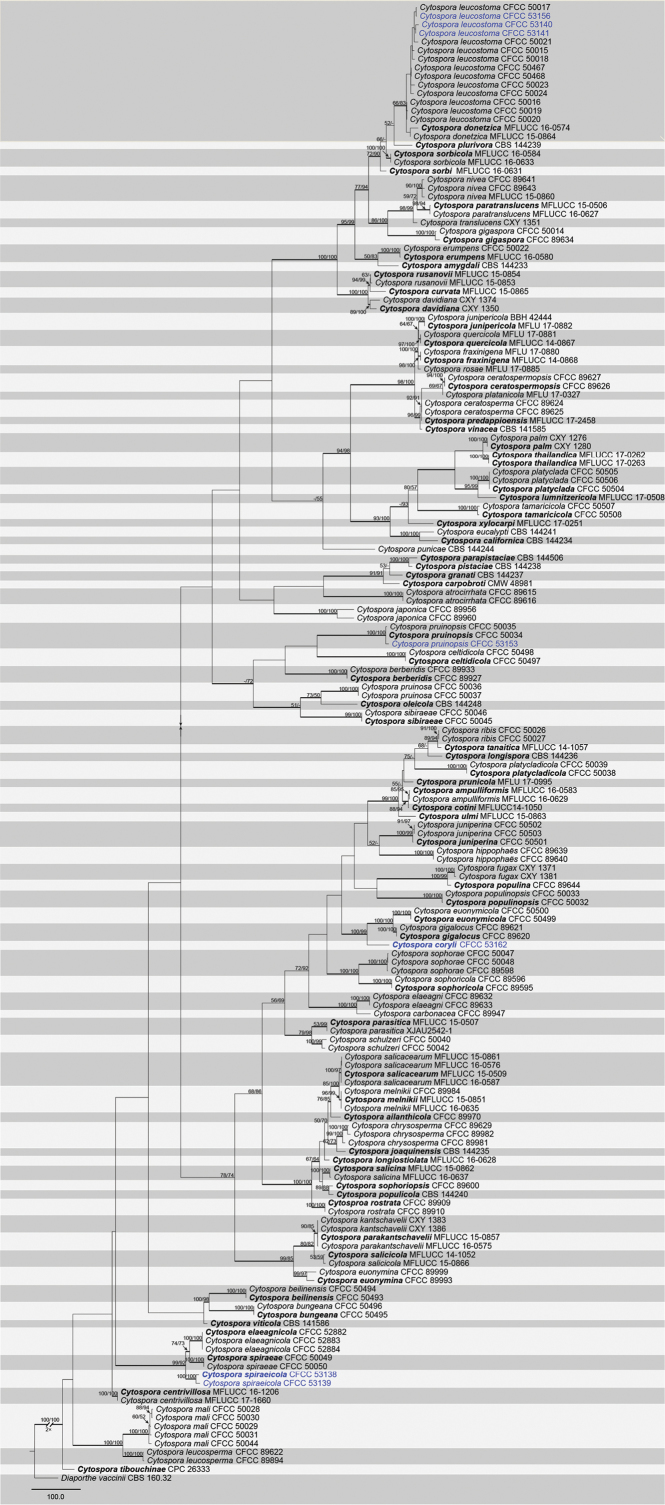
Phylogram of *Cytospora*, based on combined ITS, LSU, *act*, *rpb2*, *tef1-α* and *tub2* genes. The MP and ML bootstrap support values above 50% are shown at the first and second positions, respectively. Thickened branches represent posterior probabilities above 0.95 from the BI. Ex-type strains are in bold. Strains from the current study are in blue.

A combined matrix of six gene sequences of *Cytospora* was considered. The combined alignments matrix (ITS, LSU, *act*, *rpb2*, *tef1-α* and *tub2*) included 172 accessions (seven from this study and 165 retrieved from GenBank) and counted 3,652 characters including gaps (665 characters for ITS, 525 for LSU, 337 for *act*, 730 for *rpb2*, 771 for *tef1-α* and 624 for *tub2*), of which 2,067 characters were constant, 189 variable characters were parsimony-uninformative and 1,396 (38.22%) characters were variable and parsimony-informative. The MP analysis generated 100 parsimonious trees, the first tree of which is presented in Fig. 2 (TL = 8,029, CI = 0.345, RI = 0.804, RC = 0.278). Tree topologies of ML and BI analyses were similar to the MP tree. Based on the multi-locus phylogeny and morphology, seven strains were assigned to four species within *Cytospora coryli*, *C. leucostoma*, *C. pruinopsis* and *C. spiraeicola*, including two taxa which we describe here as new. The two isolates of *C. spiraeicola* formed a distinct and strongly supported clade (MP/ML/BI = 100/100/1) with close phylogenetic affinity to *C. elaeagnicola* and *C. spiraeae*. The strain of *C. coryli* from *Corylus mandshurica* shared a close relationship to *Cytospora euonymicola* and *C. gigalocus* with 100% MP, 99% ML, 0.99 BI supports.

### Taxonomy

#### Cytospora coryli

Taxon classificationFungiDiaporthalesValsaceae

H.Y. Zhu & X.L. Fan sp. nov.

C0298731-0C80-5D50-9972-3FA962BB480F

833820

Fig. 3

##### Etymology.

Named after the host genus on which it was collected, *Corylus*.

##### Holotype.

China, Beijing City, Mentougou District, Mount Dongling, Xiaolongmen Forestry Centre (115°27'07.00"E, 39°59'26.47"N), from branches of *Corylus mandshurica*, 17 Aug 2017, H.Y. Zhu & X.L. Fan, holotype CF 2019813, ex-type living culture CFCC 53162.

##### Description.

*Necrotrophic* on branches of *Corylus mandshurica*. ***Sexual morph***: not observed. ***Asexual morph***: *Conidiomata* pycnidial, flat, immersed in the bark, scattered to gregarious, erumpent through the surface of bark, surrounded by conspicuous black stroma walls in the margin, with multiple locules. *Conceptacle* absent. *Ectostromatic disc* grey to black, discoid, circular to ovoid, 270–340 µm in diam., with one ostiole per disc. *Ostiole* grey to black, at the same or above level as the disc surface, inconspicuous. *Locules* numerous, subdivided frequently by invaginations with common walls, circular to irregular, 1550–1710 µm in diam. *Conidiophores* hyaline, branched at the base, in the middle, approximately cylindrical with the top end acute, 15.5–18.5 × 1–2 (av. = 17 ± 1.2 × 1.1 ± 0.2, n = 10) µm, sometimes reduced to conidiogenous cells. *Conidiogenous cells* enteroblastic, phialidic, sub-cylindrical to cylindrical, 7.5–14 × 1–2 (av. = 9.3 ± 1.7 × 1.4 ± 0.2, n = 10) μm. *Conidia* hyaline, allantoid, smooth, aseptate, thin-walled, 5–7 × 1–2 (av. = 5.6 ± 0.5 × 1.4 ± 0.2, n = 30) μm.

**Figure 3.**
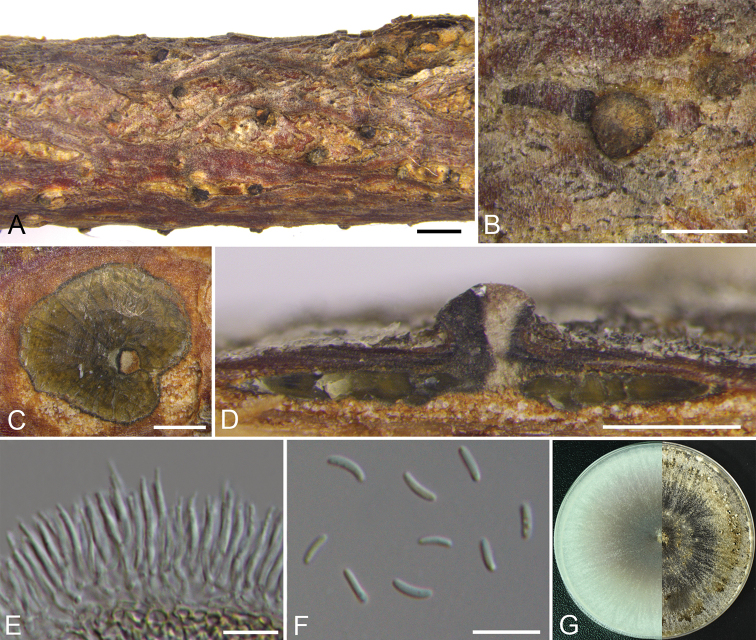
*Cytospora coryli* from *Corylus mandshurica* (CF 2019813). **A, B** habit of conidiomata on twig **C** transverse section of conidioma **D** longitudinal section through conidioma **E** conidiophores and conidiogenous cells **F** conidia **G** colonies on PDA at 3 days (left) and 30 days (right). Scale bars: 1 mm (**A**); 500 μm (**B–D**); 10 μm (**E, F**).

##### Culture characteristics.

*Cultures* are initially white with hazel at the centre, growing fast up 9 cm in diam. after 3 days, becoming honey to hazel from the edge to centre after 7–10 days. In reverse, the cultures are the same as the upper colour after 3 days, becoming cinnamon from the edge to centre after 7–10 days. *Colonies* are ﬂat, sparse at the centre and compact to the margin. *Pycnidia* distributed radially on colony surface.

##### Habitat and distribution.

Known from *Corylus mandshurica* in Mount Dongling, China.

##### Notes.

*Cytospora coryli* is associated with canker disease of *Corylus mandshurica* in China. The only strain CFCC 53162 representing *Cytospora coryli* clusters as a single lineage and appears mostly related to *C. euonymicola* from *Euonymus kiautschovicus* and to *Cytospora gigalocus* from *Juglans regia* (Fan et al. 2015a, 2020) (Fig. 2). *Cytospora coryli* differs from *C. euonymicola* by its larger locules (1550–1710 vs. 1150–1400 µm) and larger conidia (5–7 × 1–2 vs. 4.5–5 × 1 μm) (Fan et al. 2020), *C. coryli* differs from *C. gigalocus* by its smaller locules (1550–1710 vs. 1630–2180 µm) with single ostiole (one to five ostioles in *C. gigalocus*) and the larger size of conidia (5–7 × 1–2 vs. 4.6–5.6 × 0.8–1.3 μm) (Fan et al. 2015a). Based on morphology and sequence data, we describe it as a new species.

#### Cytospora leucostoma

Taxon classificationFungiDiaporthalesValsaceae

(Pers.) Sacc., Michelia 2: 264 (1881)

D20E4F61-8608-533C-815B-4F248C7DA6D6

Figs 4 , 5

Sphaeria leucostoma Pers., Ann. Bot. 11: 23 (1794)
Valsa leucostoma (Pers.) Fr., Summa Veg. Scand., Section Post. (Stockholm): 411 (1849)
Valsa persoonii Nitschke, Pyrenomyc. Germ. 2: 222 (1870)
Leucostoma persoonii (Nitschke) Höhn., Mitt. Bot. Inst. Tech. Hochsch. Wien 5: 78 (1928)[Additional synonyms in Species Fungorum.]

##### Description.

*Necrotrophic* on branches of Betulaceae, Juglandaceae and Rosaceae. ***Sexual morph***: *Ascostromata* immersed in the bark, erumpent through the surface of bark, scattered, 950–2550 µm in diam., with 8–10 perithecia arranged circularly to irregularly. *Conceptacle* absent. *Ectostromatic disc* pale grey, fusoid, 600–2150 µm in diam., with 8–10 ostioles arranged irregularly per disc. *Ostioles* numerous, dark grey to black, at the same or above the level as the disc, concentrated, arranged irregularly in a disc, 60–120 µm in diam. *Perithecia* beige with a little black when mature, flask-shaped to spherical, arranged circularly to irregularly, 270–560 µm in diam. *Paraphyses* large, broad and cylindrical with 1–4 septa, 39–78 × 5.8–8.7 (av. = 50.6 ± 13.7 × 7 ± 0.8, n = 10) μm. *Asci* free, clavate to elongate obovoid, 35–45 × 6–8 (av. = 40.4 ± 3.3 × 6.9 ± 0.5, n = 10) μm, 8-spored. *Ascospores* uniseriate to biseriate, elongate-allantoid, thin-walled, hyaline, aseptate, 7–10 × 2–3 (av. = 8.3 ± 0.9× 2.6 ± 0.2, n = 30) μm. ***Asexual morph***: *Conidiomata* pycnidial, immersed in the bark, scattered, erumpent through the surface of bark, with multiple locules and a conspicuous central column. *Central column* beneath the disc more or less conical, brown. *Conceptacle* absent. *Ectostromatic disc* buff, discoid, circular to ovoid, 190–310 µm in diam., with 1–2 ostioles per disc. *Ostioles* grey to black, at the same or above the level as the disc surface, 60–65 μm in diam. *Locules* numerous, subdivided frequently by invaginations with common walls, circular to ovoid, 700–1000 µm in diam. *Conidiophores* hyaline, branched at the base or unbranched, approximately cylindrical, 8–14 × 1–2 (av. = 11.5 ± 1.8 × 1.4 ± 0.2, n = 10) µm, sometimes reduced to conidiogenous cells. *Conidiogenous cells* enteroblastic phialidic, sub-cylindrical to cylindrical, 7–11 × 1–2 (av. = 9 ± 1.4 × 1.5 ± 0.3, n = 10) μm. *Conidia* hyaline, elongate-allantoid, smooth, aseptate, 4.5–6 × 1–2 (av. = 5.4 ± 0.3 × 1.5 ± 0.2, n = 30) μm.

**Figure 4.**
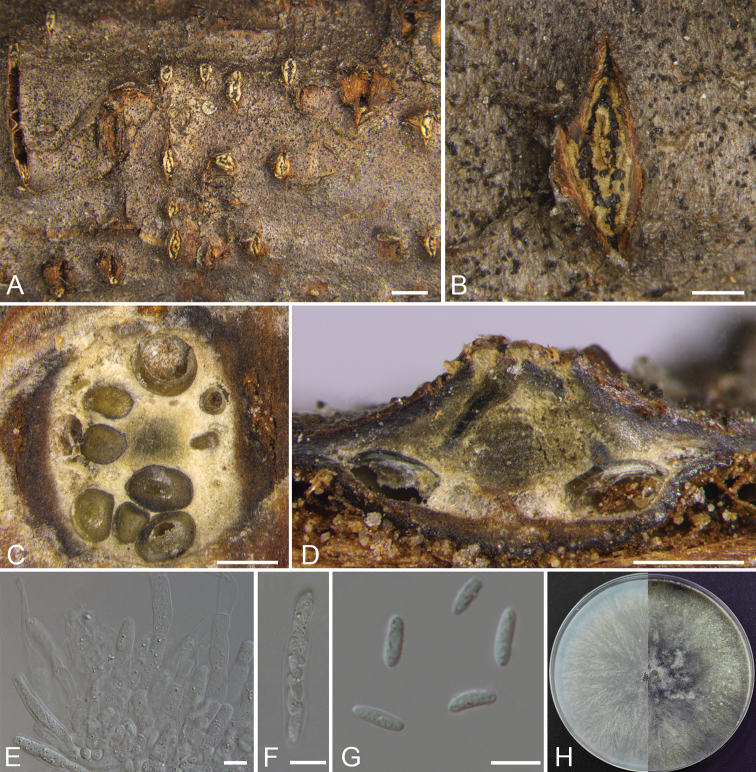
*Cytospora leucostoma* (Sexual morph) from *Prunus sibirica* (CF 2019814). **A, B** habit of ascomata on twig **C** transverse section of ascoma **D** longitudinal section through ascoma **E** asci and ascospores **F** ascus **G** ascospores **H** colonies on PDA at 3 days (left) and 30 days (right). Scale bars: 1 mm (**A**); 500 μm (**B–D**); 10 μm (**E–G**).

**Figure 5.**
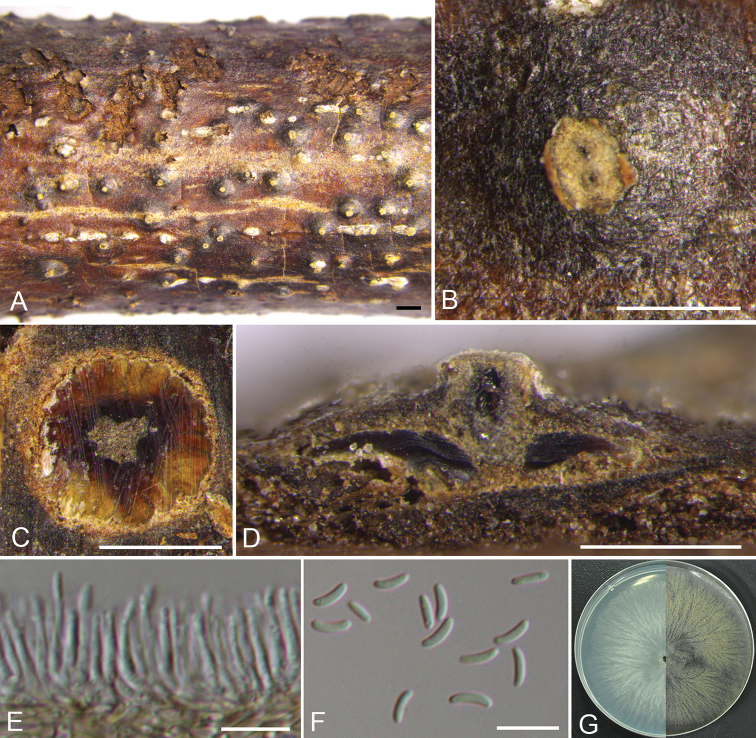
*Cytospora leucostoma* (Asexual morph) from *Juglans mandshurica* (CF 2019809). **A, B** habit of conidiomata on twig **C** transverse section of conidioma **D** longitudinal section through conidioma **E** conidiophores and conidiogenous cells **F** conidia **G** colonies on PDA at 3 days (left) and 30 days (right). Scale bars: 1 mm (**A**); 500 μm (**B–D**); 10 μm (**E, F**).

##### Culture characteristics.

*Cultures* initially are white, growing fast up to 8 cm in diam. after 3 days and entirely covering the 9 cm Petri dish after 4 days, becoming greenish-olivaceous after 7–10 days and grey olivaceous after 30 days. In reverse, the cultures are the same as the upper colour after 7 days, becoming olivaceous grey to iron grey after 30 days. *Colonies* are ﬂat with a uniform texture; sterile.

##### Habitat and distribution.

Known from several species of Betulaceae, Juglandaceae and Rosaceae around the world.

##### Materials examined.

China, Beijing City, Mentougou District, Mount Dongling, Xiaolongmen Forestry Centre (115°26'47.36"E, 39°56'06.45"N), from branches of *Prunus sibirica*, 17 Aug 2017, H.Y. Zhu & X.L. Fan, CF 2019814, living culture CFCC 53140; *ibid.* CF 2019815, living culture CFCC 53141. China, Beijing City, Mentougou District, Mount Dongling, Xiaolongmen Forestry Centre (115°29'20.52"E, 39°57'47.49"N), from branches of *Juglans mandshurica*, 17 Aug 2017, H.Y. Zhu & X.L. Fan, CF 2019809, living culture CFCC 53156.

##### Notes.

*Cytospora leucostoma* is commonly associated with canker disease of Prunoideae of Rosaceae in China (Fan et al. 2020). Morphologically, our taxa are similar to previous descriptions of *C. leucostoma* in having multi-loculate pycnidial stromata with a conspicuous black conceptacle, producing elongate-allantoid, large conidia (4.5–6 × 1–2 μm) (Teng 1963, Zhuang 2005, Fan et al. 2020). The greenish-yellow of the cultures on PDA medium from *Juglans mandshurica* is similar to descriptions of those collected from Prunoideae (Fan et al. 2020). Multigene phylogenetic analyses supported the morphological results with high support values (ML/MP/BI = 100/100/1, Fig. 2). By combining morphology and the DNA data, our isolates collected from dead branches of *Prunus sibirica* and *Juglans mandshurica* belong to this species. The current study represents a new host record of *Juglans mandshurica*.

#### Cytospora pruinopsis

Taxon classificationFungiDiaporthalesValsaceae

C.M. Tian & X.L. Fan, Mycological Progress 14(9): 74 (2015)

597FB305-1753-5013-AFB7-18158480E228

Fig. 6

##### Description.

See Yang et al. (2015).

##### Material examined.

China, Beijing City, Mentougou District, Mount Dongling, Xiaolongmen Forestry Centre (115°27'29.37"E, 39°56'47.49"N), from branches of *Ulmus pumila*, 22 Aug 2017, H.Y. Zhu & X.L. Fan, CF 2019806, living culture CFCC 53153.

##### Habitat and distribution.

Known from *Ulmus pumila* in Northern China.

##### Notes.

Yang et al. (2015) described *Cytospora pruinopsis* from cankers of *Ulmus pumila* in Shannxi Province of China. The strain CFCC 53153 clusters in a well-supported clade with high support value (MP/ML/BI = 100/100/1), based on combined multi-locus gene phylogenetic analyses (Fig. 2). Morphologically, it confirms *Cytospora pruinosa* in having a single locule and small conidia (2–4 × 1 μm) as per the descriptions of Yang et al. (2015). Phylogenetically, our isolates represent 6/771 nucleotide differences of *tef1-α* comparing with ex-type strains CFCC 50034 of *C. pruinosa*. Morphology and sequence data confirmed that our isolates represent this species.

**Figure 6.**
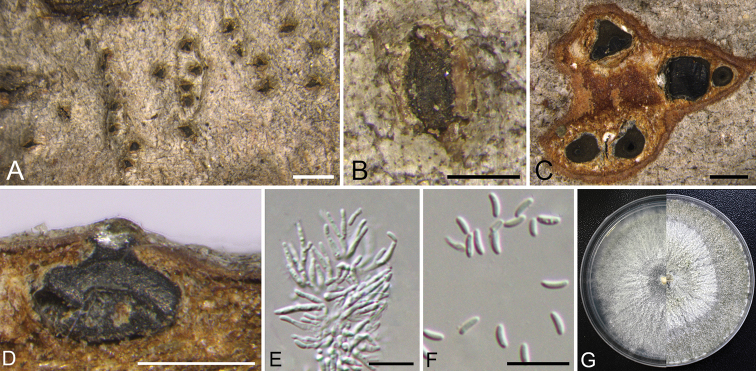
*Cytospora pruinopsis* from *Ulmus pumila* (CF 2019806). **A, B** habit of conidiomata on twig **C** transverse section of conidiomata **D** longitudinal section through conidioma **E** conidiophores and conidiogenous cells **F** conidia **G** colonies on PDA at 3 days (left) and 30 days (right). Scale bars: 1 mm (**A**); 250 μm (**B**); 500 μm (**C, D**); 10 μm (**E, F**).

#### Cytospora spiraeicola

Taxon classificationFungiDiaporthalesValsaceae

H.Y. Zhu & X.L. Fan sp. nov.

37DD8FA7-A93E-510E-BD0B-0C4C2E4998C2

833821

Fig. 7

##### Etymology.

Named after the host genus on which it was collected, *Spiraea*.

##### Holotype.

China, Beijing City, Mentougou District, Mount Dongling, Xiaolongmen Forestry Centre (115°28'28.52"E, 39°55'49.42"N), from branches of *Spiraea salicifolia*, 17 Aug 2017, H.Y. Zhu & X.L. Fan, holotype CF 2019803, ex-type living culture CFCC 53138.

##### Description.

*Necrotrophic* on branches of *Spiraea salicifolia* and *Tilia nobilis*. ***Sexual morph***: *Ascostromata* immersed in the bark, erumpent through the surface of bark, scattered, with 3–5 perithecia arranged regularly, 660–890 µm in diam. *Conceptacle* absent. *Ectostromatic disc* pale grey, usually surrounded by tightly crowded ostiolar necks, quadrangular, 240–350 µm in diam., with 5–8 ostioles arranged regularly per disc. *Ostioles* numerous, dark grey to black, at the same or above the level as the disc, concentrated, arranged regularly in a disc, 25–40 µm in diam. *Perithecia* dark grey to black, flask-shaped to spherical, arranged circularly, 210–250 µm in diam. *Paraphyses* lacking. *Asci* free, clavate to elongate, obovoid, 26–37 × 7.5–9 (av. = 33 ± 2.5 × 8.3 ± 0.9, n = 10) μm, 8-spored. *Ascospores* biseriate, elongate-allantoid, thin-walled, hyaline, slightly curved, aseptate, 8.5–12 × 2.5–3.5 (av. = 10 ± 1 × 3 ± 0.3, n = 30) μm. ***Asexual morph***: not observed.

**Figure 7.**
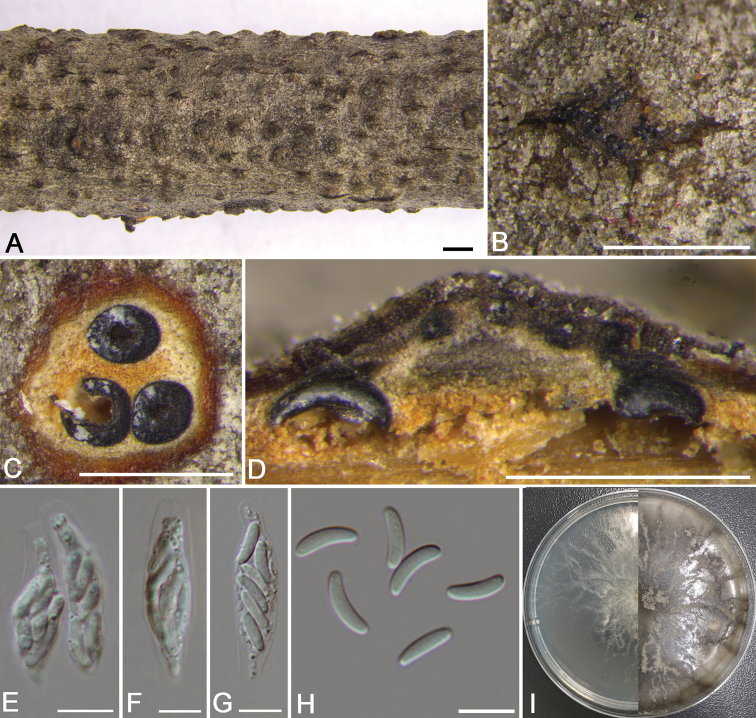
*Cytospora spiraeicola* from *Spiraea salicifolia* (CF 2019803). **A, B** habit of ascomata on twig **C** transverse section of ascoma **D** longitudinal section through ascoma **E** asci and ascospores **F, G** ascus **H** ascospores **I** colonies on PDA at 3 days (left) and 30 days (right). Scale bars: 1 mm (**A, B**); 500 μm (**C, D**); 10 μm (**E–H**).

##### Culture characteristics.

*Cultures* are white, growing up to 4 cm in diam. with irregular margin after 3 days, covering the 9 cm Petri dish after 6 days, becoming vinaceous buff to hazel after 7–10 days. In reverse, the cultures are the same as the upper colour after 3 days, becoming isabelline to umber after 7–10 days. *Colonies* are felty with a heterogeneous texture, lacking aerial mycelium.

##### Habitat and distribution.

Known from *Spiraea salicifolia* and *Tilia nobilis* in Mount Dongling, China.

##### Additional material examined.

China, Beijing City, Mentougou District, Mount Dongling, Xiaolongmen Forestry Centre (115°29'20.49"E, 39°57'47.43"N), from branches of *Tilia nobilis*, 17 Aug 2017, H.Y. Zhu & X.L. Fan, CF 2019804, living culture CFCC 53139.

##### Notes.

*Cytospora spiraeicola* is associated with canker disease of *Spiraea salicifolia* and *Tilia nobilis* in China, with characteristics similar to *Cytospora elaeagnicola* and *C. spiraeae* in phylogram (Fig. 2). Morphologically, it differs from *C. spiraeae* by the smaller perithecia (210–250 vs. 270–400 µm in diam.) and longer ascospores (8.5–12 × 2.5–3.5 vs. 7–8 × 2–2.5 µm) (Zhu et al. 2018a). Phylogenetically, *C. spiraeicola* (CFCC 53138) differs from *C. elaeagnicola* (CFCC 52882) by ITS (8/665), *rpb2* (44/730), *tef1-α* (75/771) and *tub2* (42/624) and *C. spiraeae* (CFCC 50049) by ITS (4/665), *rpb2* (38/730), *tef1-α* (63/771) and *tub2* (44/624) (Zhu et al. 2018a, Zhang et al. 2019). Therefore, we describe it as a novel species.

## Discussion

In the present study, seven specimens were collected from symptomatic branches and twigs associated with canker disease. Four *Cytospora* species were isolated from six tree hosts of Betulaceae, Juglandaceae, Rosaceae, Tiliaceae and Ulmaceae, which include two known species (*Cytospora leucostoma* and *C. pruinopsis*) and two novel species (*C. coryli* and *C. spiraeicola*). This study represents an investigation of *Cytospora* species associated with canker disease in Mount Dongling of China and included a comprehensive analysis of DNA sequence data to compare the novelties with known *Cytospora* species.

In a previous study, Zhu et al. (2018a) described *Cytospora spiraeae* from *Spiraea salicifolia* in Gansu Province of China during an investigation of forest pathogens of three hosts. Compared to the new species *Cytospora spiraeicola*, *C. spiraeae* has larger perithecia (270–400 vs. 210–250 µm) in diam. and shorter ascospores (7–8 × 2.5–3.5 × 8.5–12 vs. 2–2.5 µm). These morphological deviations are in line with the combined phylogenetic analyses which resolved *C. spiraeicola* as a unique lineage, highly supported. Besides this, the only strain of *C. coryli*, closely related to *C. euonymicola* and *C. gigalocus*, was distinguished by its different size of multiple locules and conidia (Fan et al. 2015a, 2020).

This study focused on *Cytospora* species in Mount Dongling of Beijing (China), which is considered as an attractive location with a high richness of fungal species (Guo et al. 2008, Zhu et al. 2018b, 2019). We hope that the descriptions and molecular data of *Cytospora* in this study could provide a resource for future studies in this genus and lay the foundation for the future canker disease caused by *Cytospora* species.

## Supplementary Material

XML Treatment for Cytospora coryli

XML Treatment for Cytospora leucostoma

XML Treatment for Cytospora pruinopsis

XML Treatment for Cytospora spiraeicola
